# A Great Benevolent Enterprise

**Published:** 1856-04

**Authors:** 


### A GREAT BENEVOLENT ENTERPRISE.

The Board of Managers of the "Pennsylvania Hospital for the Insane," at Philadelphia, have determined upon the erection of a new and much larger building than the old, which, it is estimated, will cost two hundred and fifty thousand dollars, to be raised by private subscription. Over one hundred and fifty thousand are already subscribed.

We have heretofore claimed for the medical profession, the meed of being always first and most liberal in these great works of charity, which is proved in this undertaking; about seven thousand being donated by fifteen medical men.

In a letter from Dr. Kirkbride, the Superintendent, to one of the editors, he says: "We have a large undertaking on hand, and which we commence immediately."

In such a heaven-born enterprise we wish them success, and trust that an example so liberal and christian, may be followed by the citizens of other cities.

